# Voting Ensemble Approach for Enhancing Alzheimer’s Disease Classification

**DOI:** 10.3390/s22197661

**Published:** 2022-10-09

**Authors:** Subhajit Chatterjee, Yung-Cheol Byun

**Affiliations:** 1 Department of Computer Engineering, Jeju National University, Jeju 63243, Korea; 2 Department of Computer Engineering, Major of Electronic Engineering, Institute of Information Science & Technology, Jeju National University, Jeju 63243, Korea

**Keywords:** Alzheimer’s disease, deep learning, classification, ensemble learning, MRI data

## Abstract

Alzheimer’s disease is dementia that impairs one’s thinking, behavior, and memory. It starts as a moderate condition affecting areas of the brain that make it challenging to retain recently learned information, causes mood swings, and causes confusion regarding occasions, times, and locations. The most prevalent type of dementia, called Alzheimer’s disease (AD), causes memory-related problems in patients. A precise medical diagnosis that correctly classifies AD patients results in better treatment. Currently, the most commonly used classification techniques extract features from longitudinal MRI data before creating a single classifier that performs classification. However, it is difficult to train a reliable classifier to achieve acceptable classification performance due to limited sample size and noise in longitudinal MRI data. Instead of creating a single classifier, we propose an ensemble voting method that generates multiple individual classifier predictions and then combines them to develop a more accurate and reliable classifier. The ensemble voting classifier model performs better in the Open Access Series of Imaging Studies (OASIS) dataset for older adults than existing methods in important assessment criteria such as accuracy, sensitivity, specificity, and AUC. For the binary classification of with dementia and no dementia, an accuracy of 96.4% and an AUC of 97.2% is attained.

## 1. Introduction

The anatomical features of the brain are studied using magnetic resonance imaging (MRI), which has a high spatial resolution and can contrast soft tissue. It is well recognized that, in comparison to other imaging modalities such as computed tomography (CT) and positron emission tomography (PET), magnetic resonance imaging (MRI) often has fewer health concerns. In the past few decades, significant advancements have been made in evaluating brain injuries and using MRI to study alterations in the brain. The most prevalent kind of alterations in the brain is Alzheimer’s Disease (AD), which is a disorder of brain functions. It impairs patients’ knowledge, planning, thinking, and judgment and makes it harder for them to carry out daily tasks.

The introduction of machine learning methodologies has dramatically increased the amount of research being conducted on AD. Accurate identification and classification of ill participants and the healthy subjects around them are crucial for identifying diseases such as AD. For more precise diagnoses, a lot more data is needed. To help doctors make an early diagnosis of Alzheimer’s, computer-aided diagnosis (CAD) systems employ the built-in patterns of neuroimaging data and machine learning algorithms.

Although there is presently no cure for Alzheimer’s, early detection can help the disease’s progression. Therefore, it is crucial to find it when the condition is still in the initial stage. There is no unambiguous way to demonstrate AD clinically. However, research has produced some excellent findings using computer-aided methods. Alzheimer’s disease is the most well-known and familiar in older people’s lives. According to Ron et al. [1], 26.6 million people worldwide are partially or fully affected by Alzheimer’s disease and expected the value will double by 2050. Over 60% of all Alzheimer’s disorders are caused by Alzheimer’s disease [2], a chronic neurological ailment. Alzheimer’s disease is far more likely to develop with age. Diagnostic services are restricted mainly to tertiary care hospitals in population centers in many middle-income countries because of the rapidly aging global population.

Several high-dimensional classification techniques exclusively categorize AD and healthy controls using structural MRI brain images. The recently high dimensional classification was proposed by [3] for detecting AD or mild cognitive impairment (MCI). Historical data, clinical presentation, and behavioral observations are typically used to diagnose AD. It might be challenging to make an objective and reproducible diagnosis when specialists working in memory clinics exhibit shockingly low levels of diagnostic agreement with one another [4]. Alternatively, more opinions should be sought from primary care services to improve the way of treatment because of the lack of AD specialists in many parts of the world. Improved adaptation of medical diagnosis leads to a precise medical environment. Hence, finding ways to leverage experts’ intelligence better is essential. Our framework is an effective strategy to assist existing or new health professionals with insufficient AD and make clinical diagnoses [5,6]. The main contributions of this study are as follows:

- Our proposed voting ensemble method for classifying AD is more accurate than other conventional ensemble approaches, pointing to a fresh approach to improving Alzheimer’s disease meticulous care.
- To modify the data format in a consistent manner, we have several data pre-processing procedures.
- A voting ensemble method is proposed that combines predictions of multiple classifiers, and the predictions for each label are summed. The majority vote has been taken for predicting the label.
- Our suggested voting ensemble model performance compared with the conventional models and different combinations of ensemble models.

## 2. Related Works

Clinical evaluation is complemented by identifying physical changes in the brain, which is becoming more and more crucial. Researchers are focusing on imaging approaches to evaluate AD to detect it early. To identify early AD, machine learning is crucial to advancing treatment. This method produced classifiers for diagnosing AD using picture data and clinical assessments. Many researcher have been doing study on precise treatment and improve the medical diagnosis [7,8,9,10,11,12,13]. These investigations have revealed the important structural variations between the healthy brain and the brain affected by AD in areas such as the entorhinal cortex and hippocampal regions. Changes in the brain’s tissues can explain the erratic behaviors of AD patients [14,15]. Ye et al. [16] proposed a semi-supervised method for classifying AD by employing a non-linear manifold learning technique for high dimension reduction and, with the Laplacian SVM classifier, achieved an AUC value of 0.73.

In recent studies in medical image classification, Bhardwaj et al. [17] took advantage of a convolution neural network by using two transfer learning models for the classification of Alzheimer’s disease. Their proposed inception-ResNetV2 CNN model was trained with their data and achieved an accuracy of 97.3%. Ensemble learning concept employed by [18] for dementia classification with voting classifier and achieved a better result than other well-known machine learning models. The authors [19] proposed a computer-aided diagnosis (CAD) system with five stages. In the first stage, images were preprocessed and segmented into gray matter (GM), white matter (WM), and cerebrospinal fluid (CSF). In the second stage, similarity matrices were constructed using the GM segmented ROIs, and statistical feature extraction took place in the third stage. The functional activities questionnaire (FAQ) and statistical features were integrated with clinical data in the final two rounds, and to classify the data between AD and normal groups, an SVM as a classifier was employed. In [20], a three-dimensional displacement-field calculation was utilized to categorize AD patients and elderly individuals in good health. Three techniques—Student’s *t*-test, Welch’s *t*-test, and the Bhattacharyya distance were used to select features. A SVM classifier was used to classify the data, and the classification accuracy was 93.05%. Measurements of cortical morphology from individual gray matter images were used to construct a structurally connected network. A statistical feature generation approach based on histogram-based feature generation methods has been proposed to represent the statistical patterns of interconnected networks from high-dimensional spaces to low-dimensional feature vectors. Finally, using the SVM classifier, the authors [21] secured an accuracy of 84.1%.

Kloppel et al. [22] proposed to use gray matter from T1-weighted MR scan pictures to classify AD using linear support vector machines. The Authors [23] have employed both the SVM binary classification job and multi-class classifier to detect AD from MRI images using dimensional reduction, formulation modifications, and PCA. Vemuri et al. [24] used to create three models for structural MR image-based support vector machine (SVM)-based diagnosis of AD in individual participants. In [25], the purpose of diagnosing AD, a random forest classifier, is used to classify MRI and PET imaging data using a multi-modality classification framework based on similarities produced from random forests. The proposed method by [26], which employs the gray-level co-occurrence matrix (GLCM) method for AD classification, results in an average testing accuracy of 75.71%.

In [27], a feature-based automatic computer-aided diagnostic (CAD) method for AD identification using structural magnetic resonance imaging data. As a classifier, SVM is utilized to categorize patients into AD and healthy patients. With 92.48% accuracy, the suggested method performs better than other models. In this study, [28], an attempt has been made to use shape-based Laplace Beltrami (LB) eigenvalue characteristics and machine learning approaches to examine the corpus callosum (CC) shape changes. Compared to the other two widely used classifiers, SVM and NB as well as K-nearest neighbor (KNN) achieved an accuracy of 93.37% when classifying the significant features based on Information Gain (IG) ranking.

In order to combine classifiers with an enhanced prediction of data, ensemble learning makes use of a collection of decision-making systems that employ a variety of methodologies. A common method used by ensembles is a hard voting ensemble, which predicts the class that will receive the most votes based on the cumulative forecasts of all classifiers. In healthcare-related fields, ensemble learning has been used to increase the accuracy of AD prediction by utilizing the clinical experience of typical doctors [29,30]. It might be applied in primary care settings where access to specialists is constrained.

## 3. Methodology

**Figure 1:**
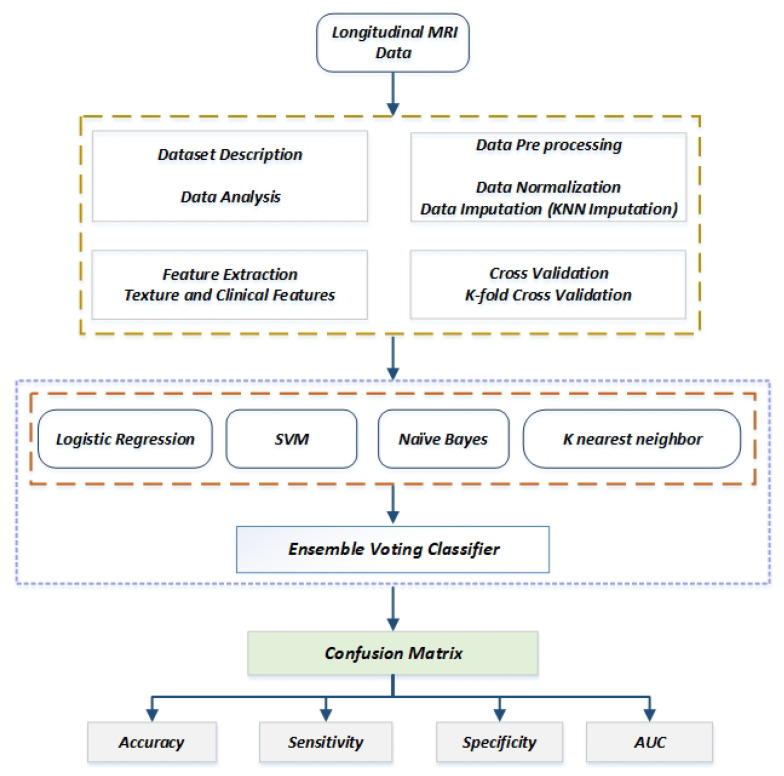
Workflow of the proposed framework.

The method is split into several halves, and the first half includes dataset, data preprocessing, and feature extraction. The second half consists of a different machine learning classifier with a proposed voting ensemble approach. The proposed methodology is elaborated in Figure 1, and each half will be elaborately discussed in the coming subsections.

### 3.1. Dataset

The data we have used in this study is MRI data created by the Open Access Series of Imaging Studies (OASIS) project [31], which is available on their website as well. This well knows dataset is used in machine learning for precision medicine and diagnosis of patients with and without dementia. The OASIS aims to provide brain MRI data sets publicly accessible to the scientific community. The Research Center manages an enormous volume of longitudinal and cross-sectional brain MRI data from both healthy and mentally ill individuals. The cross-sectional category comprises information on 150 patients with ages ranging from 18 to 96 years, while the longitudinal dataset includes several scans of each subject over a while. Diagnosis of AD needs a proper medical analysis which includes the clinical dementia rating (CDR) and mini-mental state examination (MMSE); one can assess the risk factor for AD. Risk factors are studied in relation to the participants, as stated in Table 1.

For our experiment, we have used longitudinal MRI data in CSV format. For acquiring images using a 1.5-T Vision scanner (Siemens, Erlangen, Germany), three to four distinct T1-weighted magnetization prepared rapid gradient-echo (MP-RAGE) [32] images were collected for each subject during a single imaging session. Figure 1 in [31] illustrates a typical image as an example. The CDR scale was used to determine the status of dementia according to the CDR value.

#### 3.1.1. Dataset Description

Dataset has some important columns which hold a value for this research. The experiment was carried out with a few key components, including that each patient is right-handed and that they were all scanned at least once. Throughout the study with the patients, 72 patients were categorized having no dementia, whereas 64 of the patients were classified as having dementia at the time of their first visits and remained in this category for the duration of the study. Fourteen participants who were first categorized as not having dementia were later labeled as having dementia during a subsequent visit, which means they were classified as converted.

#### 3.1.2. Data Preprocessing

**Figure 2:**
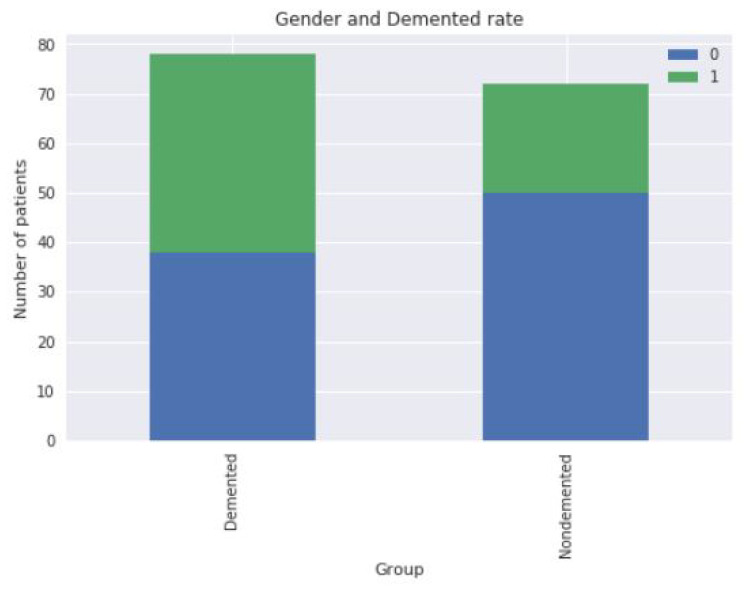
Rate of dementia for men and women.

At first, we have replaced data converted to a person with dementia, as the study proposed a classifier to predict the patients with and without dementia. After that, we removed useless columns that were not needed for our research. We have a total of 15 columns or features in the dataset, and from there, we removed the 3 unnecessary columns for our final dataset. We will use a label encoder for our binary attributes, sex, and our classes, just that it will do the one-hot encoding for the supplied column. According to the data [31] we have used in this experiment, we can visualize that from Figure 2, men are more to have dementia than women. Which indicates that patients with dementia, patients without dementia, and 1 = M (male), 0 = F (female).

#### 3.1.3. Data Analysis

**Figure 3:**
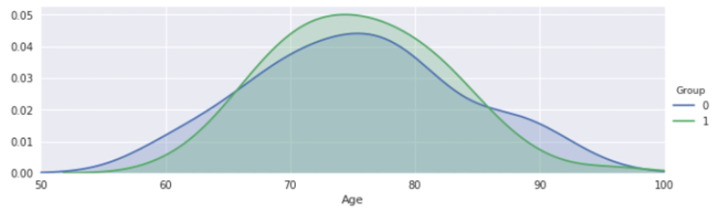
Age factor of men and women.

This section has examined the connection between each MRI test finding and the patient’s dementia. In order to assume correlations prior to data extraction and analysis, we carried out this data analysis technique to explicitly declare the relationship of data through a graph. We go deeply into the data to comprehend its internal value representation, which may aid in our understanding of the data’s nature and help us choose the best analysis strategy for the model in the future. Age factor is the key point in brain disorders or dementia diseases. In [33,34,35,36], we can understand that mainly older people affected by dementia, as visualized in Figure 3 that the patient’s with dementia group had a higher percentage of people aged 70 to 80. Where 0 indicates no dementia and 1 shows the dementia group. We hypothesize that individuals with that type of disease have a poorer rate of survival and that just a handful of them are 90 years old.

#### 3.1.4. Data Imputation

To train the model with real-world data sets facing a big problem, they have missing values, frequently represented as blanks, NaNs, or other placeholders. However, scikit-learn estimators cannot be used with these data sets since they presuppose that all values in a matrix are numerical and have significance. Discarding rows and filling in the blanks in columns is a fundamental technique for exploiting incomplete dataset. However, this comes at the cost of potentially crucial data loss. Impute the supplied values, or deriving them from the known portion of the data, is a superior course of action. With the help of the mean, median, or most frequent value of the row or column where the missing values are detected, the Imputer class offers fundamental methods for imputing missing values. Additionally, this class supports several encoding for missing values.

#### 3.1.5. Feature Selection

**Figure 4:**
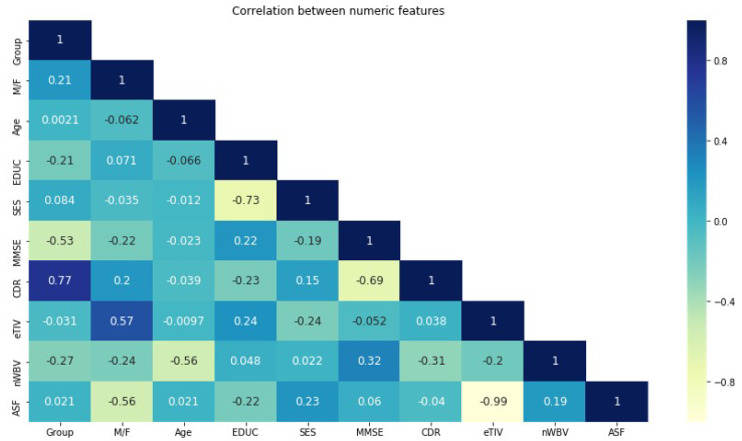
The correlation between numerical features.

Another crucial method for dimensionality reduction is feature selection which chooses the most discriminative characteristics while also removing the redundant ones [24,37]. Feature selection is one of the first and most crucial tasks in any machine learning activity. We used the Pearson correlation coefficient [38] to choose the experiment’s input variables. The correlation coefficients for several variables are shown in a correlation matrix table. The variables are displayed in rows and columns of a correlation matrix. In order to determine whether certain variables are helpful or associated with others, the first step in any factor analysis is to create a correlation plot of all the variables. The correlation between numerical features is displayed in Figure 4. However, should be carefully taken into account throughout the feature selection process because the disease frequently affects spatially contiguous regions rather than isolated voxels. The most crucial factor that will enhance classification performance is feature selection. More crucially, due to the possibility of data overfitting, as the feature subset chosen by correlation algorithms depends on the training data set, it might not be the best choice for the test data set. As a result, ensemble learning was utilized as a generic meta learning technique to combine the predictions of various classifiers in order to increase the generalizability and robustness of individual classifiers rather than creating a single classifier with an ideal subset of features. By balancing the best feature selection with the risk of data overfitting to a particular population, this ensemble technique can reduce the potential problem of data overfitting and achieve strong generalization ability.

### 3.2. Different Models Used in Classification

We will go into detail about the suggested classification algorithms in this section. Here, binary classification of AD was carried out using a voting ensemble technique. The classifiers chosen were SVM, logistic regression, naive Bayes, and K nearest neighbor. They were all utilized to create a voting ensemble model and trained to divide data into normal and AD groups. The groups were split up into AD and normal patients. Since most texture-based classifiers, including SVM, logistic regression, naive Bayes, and K nearest neighbor, perform well on binary classification, splitting output classes provides binary class classification. The following subsections discuss the chosen classifiers.

#### 3.2.1. Logistic Regression

The classification method known as logistic regression is used to categorize observations into a separate set of classes [39]. Logistic regression alters its output using the logistic sigmoid function to produce a probability value that can be translated into two or more discrete classes, in contrast to linear regression, which produces continuous number values. A supervised classifier in machine learning is logistic regression. The prediction label can only take discrete values for a specific set of features in a classification issue.

#### 3.2.2. Support Vector Machine

A support vector machine (SVM) is a supervised machine learning classifier used in the machine learning research field. Corinna et al. [40] in 1995 proposed this algorithm particularly for classification problems [41]. A support vector machine produces the borders between the data. Support vector machines help with vector space management. As a result, the boundary is a separating hyperplane. The algorithm aims to identify the best decision boundary for classifying n-dimensional space into groups so that additional data points can be added during the training phase and placed in the proper class. A hyperplane is the name of this decision boundary. The method compares testing data with the hyper-plane after training and then reports the class to which the data belongs.

#### 3.2.3. Naive Bayes

A probabilistic model called a naive Bayes classifier [42] is used to distinguish between various objects based on specific features. The phrases naive and Bayes combine to form the phrase naive Bayes algorithm. Since it presumes that the occurrence of one feature is unrelated to the incidence of other traits, it is naive. Bayes because the Bayes theorem serves as the foundation of the classifier. A naive Bayes classifier can be trained with less data when there is conditional independence since it frequently converges more quickly than discriminative models such as logistic regression. The naive Bayes classifier nonetheless performs better in actual use despite the fact that this assumption is frequently untrue in the real world.

#### 3.2.4. K Nearest Neighbor

A supervised classification system called KNN [43] sorts new data points according to the nearby ones. KNN, a straightforward non-parametric technique, is utilized to categorize the feature sets. It determines the set of k training items that are determined to be most similar to the test object. The test item is also given a class by KNN based on the neighborhood relation. It is easy to compute and effective. It is also a memory-based classifier strategy that models the dataset without the need for additional parameters. When classifying items, there are three crucial tasks to do. After all the previous data has been recorded, a unique data point is categorized using the KNN algorithm based on similarity. This indicates that as new data comes, it may be quickly categorized into a suitable category.

#### 3.2.5. Ensemble Voting Classifier

Instead of using a single model as a classifier, an ensemble technique is hired with the motive of combining a group of different classifiers and works as a single classifier. Ensemble approaches perform better than individual machine learning algorithms because they are more adaptive and flexible. All classifiers influence the ensemble’s ultimate outcome because these approaches employ a weighted average of all method predictions. A voting classifier is a type of computational ensemble method for combining predictions from various models. A voting classifier is a technique for predicting the final output class label based on the maximum vote majority after aggregating the results of each individual classifier. The primary idea behind it is that a single unified ensemble model is constructed rather than creating different classifier models and calculating the accuracy rate for each one separately. By aggregating the majority of predicted class votes acquired for each class label, this ensemble model, which the separate models train, is in charge of predicting the output class label. The predictions made by the models employed in the voting ensemble are accurate. Compared to using a single model in that ensemble, it is quite productive and aids in improving the model’s performance.

**Figure 5:**
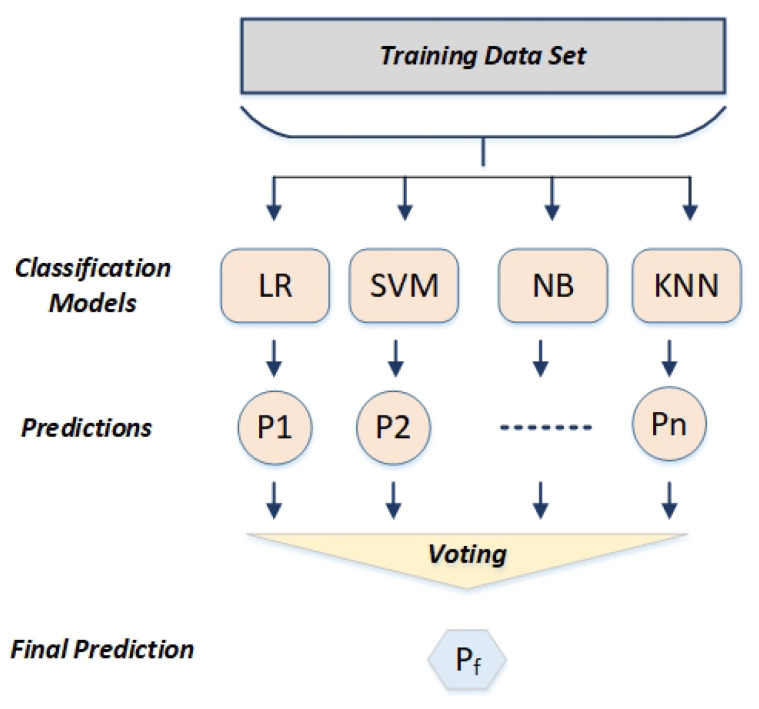
Ensemble voting classifier.

An ensemble machine learning model that integrates the predictions from various other models is known as a voting ensemble technique, as shown in Figure 5. In our study, we used the hard voting technique, which predicts the class with the highest votes based on the combined predictions of each classifier.

## 4. Results

The following subsections provide and explain the specifics of the findings from the experiments carried out for this study.

### 4.1. Implementation Environment Details

For our experiment’s classification task, we have used the system specification shown in Table 2. To implement and conduct a series of experiments python used as core language. Other libraries are required at the beginning, they are tensorFlow, Keras, Sklearn, Matplotlib, NumPy, and pandas.

### 4.2. Evaluation Parameters

The proposed system evaluated the performance of AD classification using a confusion matrix containing the actual and predicted values. The AD classification confusion matrix, with two results. TP stands for true positive or the number of AD patients accurately identified as having AD. FP stands for false positive or the number of non-AD patients diagnosed with AD. False negative (FN) refers to the number of AD patients mistakenly categorized as non-AD. TN stands for true negative or the proportion of participants who were accurately categorized as non-AD subjects. The area under the curve (AUC) is also selected as the main performance metric in our research. The performance under the curve of a binary classifier is measured by the area. Medical diagnostics for non-life-threatening terminal diseases, such as most brain disorder disorders, must have a high true positive rate to detect all Alzheimer’s patients quickly. However, we also want to ensure that the false positive rate is as low as is practical since we do not want to wrongly diagnose a healthy adult with a mental condition and begin medical therapy. AUC therefore appeared like the perfect option for a performance metric. The voting ensemble classifier and all of the base classifiers are assessed using the four metrics AUC, accuracy as shown in Equation (1), sensitivity as shown in Equation (2), and specificity as shown in Equation (3).

(1) $$Accuracy=\frac{\left(TP+TN\right)}{\left(TP+TN+FP+FN\right)}$$

(2) $$Sensitivity=\frac{TP}{\left(TP+FN\right)}$$

(3) $$Specificity=\frac{TN}{\left(TN+FP\right)}$$

### 4.3. Performance Evaluation

For our experiment, 70% of the data is used for training, and the remaining 30% is used for testing. We have a total of 150 unique patients, 105 were used in training, and 45 were used for testing. Cross validation was used to train and validate the models for efficient classification performance. To confirm the efficacy of the features gathered, four classifiers were utilized. Logistic regression, naive bayes, SVM, and KNN were the classifiers utilized. All classifiers were trained using 5-fold cross-validation, which separates the data into five equal halves. To increase the robustness of classification, it is better to divide the task into much smaller groups and repeat the method more times.

**Figure 6:**
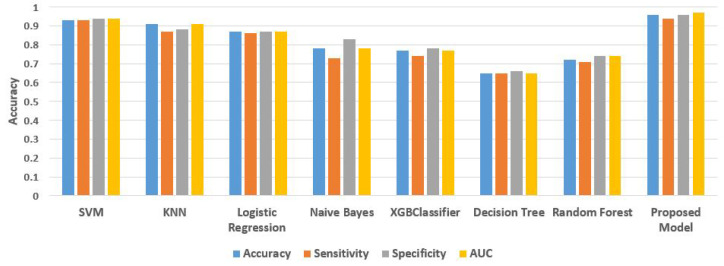
The accuracy of different model for classification.

On the OASIS dataset, Table 3 displays the binary-class classification performance of our suggested voting ensemble model. We used the four classifiers SVM, KNN, naive Bayes, and logistic regression and ensemble them using the voting ensemble method because they outperformed all other models in terms of performance. Machine learning models can function quite well on their own, but in this study, we found that merging a number of the best-performing ML models can lead to even better outcomes. When evaluated using several evaluation metrics, the suggested model outperforms the individual models. The suggested voting ensemble model accuracy is 0.96; its sensitivity, specificity, and AUC are 0.94, 0.96, and 0.97, respectively. Figure 6 compares the performance of the classification outcomes of the proposed voting ensemble model and the four traditional classification models. It can be noticeable that SVM and KNN have shown exceptional performance for classification. The dataset should be initialized, and then k-nearest neighbors should be used. The distance between the neighbors needs to be calculated next. The classification of output labels must be completed last. The value of k is fixed by iteration and is dependent on the classification accuracy. For the classification in this analysis, the number of neighbors is set at 5, and it was chosen using the trial-and-error method. We discovered that setting the classifier’s value to 5 gave us the best results.

**Figure 7:**
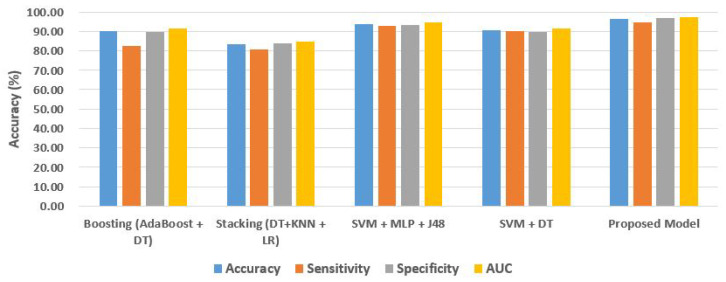
The accuracy of different ensemble model for classification.

Table 4 displays the outcomes for binary class categorization using different ensemble models. It specifies that results obtained using different ensemble classification methods on the same data. Table 4 makes it clear that our suggested ensemble model outperforms other ensemble models in terms of accuracy. In Figure 7, five ensemble strategies for categorizing people as insane or not were compared. When compared to previous ensemble approaches, which need a lot of resources and take a long time, our suggested method computes binary classification accuracy with a significant margin of error.

### 4.4. Classification Result Discussion

Data entered the preprocessing stage at the start of our study. The data will first be standardized and encoded to numerical values by applying various mathematical models. The missing values will be filled in using KNN imputation. The K-fold cross-validation approach was used to divide the data into train and test blocks. For the evaluation of our system, a 5-fold cross-validation strategy is used. The goal of choosing cross-validation is to improve the classifiers hyperparameters. Additionally, cross-validation is used to generalize results to different datasets in order to build prediction models. Conventional classification techniques logistic regression, naïve bayes, SVM, and KNN have five different estimators with different parameter tuning. These estimators are merged in a list and combined in a voting ensemble classifier. Next, the classification based on voting strategy will be proposed and applied to classify the data.

**Figure 8:**
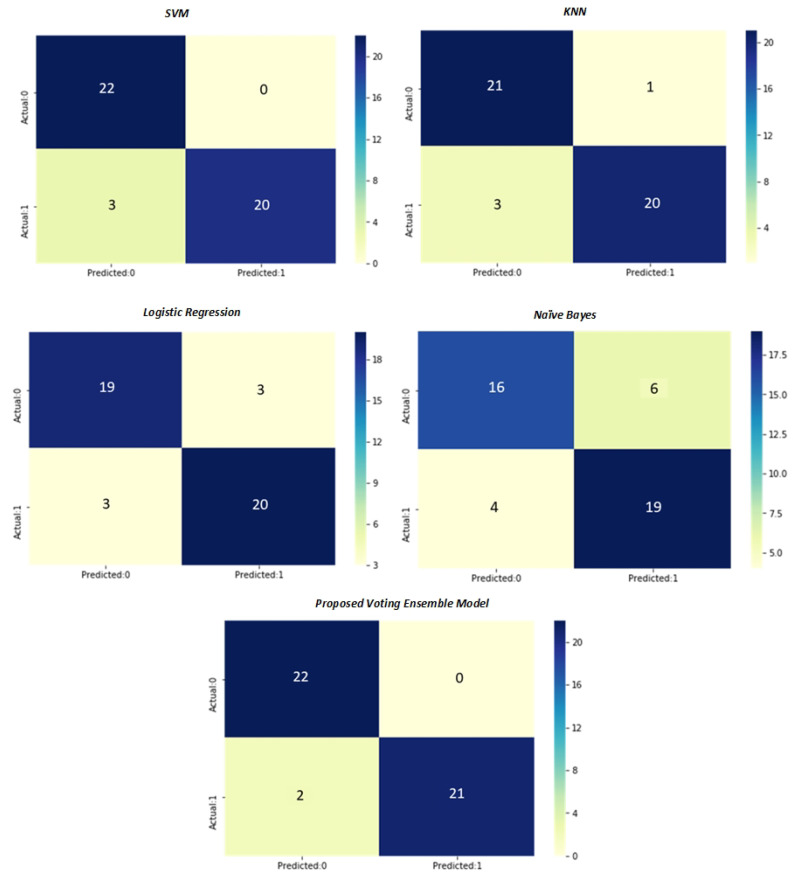
Confusion matrix of AD classification.

In Figure 8, we show the confusion matrix of SVM, KNN, logistic regression, and proposed voting ensemble model performance for the MRI data. The *x*-axis represents the predicted categories, and the *y*-axis represents the actual categories. As shown from the figure, we have used 30% of the total data for testing purposes, so 45 patients are used for testing. The figure depicts that the SVM classifier’s total of three patients had inaccurately predicted, whereas the KNN classifier’s total of four patients had inaccurately predicted; the logistic regression classifier’s total of six patients had inaccurately predicted. Although there is still space for improvement, it is clear from the figure that the suggested voting ensemble model is very accurate in classifying these groups and total oftwo2 patients had inaccurately predicted.

**Figure 9:**
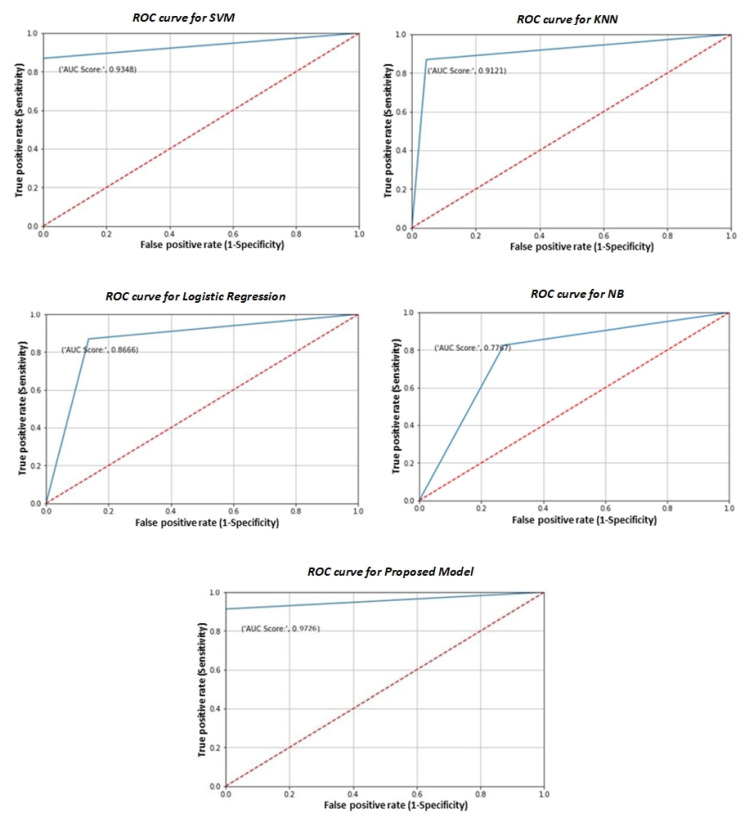
ROC curves of AD classification.

The AUC is an overall indicator of a classifier’s performance across all decision criteria. Receiver operating characteristics (ROC) curves are used to determine which of the used models predicts the class best and most beneficial of its application. We use a ROC plot to see how well a model performs between sensitivity and specificity. The capacity to appropriately identify classes as belonging to the positive class is referred to as sensitivity. Specificity is the ability to identify entries that belong in the negative category accurately. The true positive rates are deployed in a blue line, and false positive rates are plotted in a red line. The better a model is, the closer its AUC is to 1. As a result, models with higher AUC values are favored over those with lower AUC values. Figure 9 is showing different models ROC curve results.

The suggested voting ensemble model produces superior outcomes, with an AUC value of 97%. The outcome of our suggested voting ensemble model was significant. A clinician should be able to enter MRI results, patient biographical information, and other details for a patient. Our model ought to make it easier for them to recognize dementia. However, based on data from other people who have those qualities, we can assume that a person with a particular set of traits is more likely to be diagnosed with dementia. Although it is crucial to remember that Alzheimer’s is a complicated mental condition, we cannot entirely rely on ML algorithms to reach a diagnosis.

## 5. Discussion

This study investigated binary classification approaches using longitudinal MRI data for individuals with and without dementia. The patient set was divided into two groups: a training set and a testing set, to assess and compare the performances of the proposed method with other ensemble methods. A grid search on the training set was used to identify the ideal parameter values for each technique. The classifier was then trained using those values and the training group, and its performance was assessed using the testing set. We could obtain accurate estimations of how well each strategy performed in this way.

## 6. Conclusions

We used analysis of brain longitudinal MRI data to provide a quick diagnosis of AD. AD diagnosis in its early stages may benefit significantly from our suggested voting ensemble approach. We think the suggested model can be applied successfully to additional classification issues in the medical area, even if it has only been evaluated on the AD dataset. Additionally, we suggest voting ensemble-based classification models that are trained using a variety of fundamental machine learning models, including the LR, NB, KNN, and SVM. One of the significant advances is the implementation of a voting ensemble-based technique, and the effectiveness of various classifiers is assessed in terms of accuracy and AUC. Additionally, measures are employed to compare these classifiers, such as the ROC curves are used to compare the proportion of true positives to false positives at various threshold settings. The AUC is an overall indicator of a classifier’s performance across all decision criteria. According to experimental findings, voting ensemble-based classifiers outperform single classifier models LR, NB, KNN, and RF in terms of accuracy and AUC.

A sophisticated model cannot be entirely implemented due to the dataset’s size. The ranges of each group’s test value are poorly classified, despite the fact that the nature of each feature is clear to see. At least two variables limit the current study. First, there is insufficient data to allow for accurate classification results. Second, we do not examine the classifier’s capacity to differentiate several stages of dementia simultaneously, i.e., multi-class classification of AD, MCI, and healthy controls, as we only analyze binary classification between patients with and without dementia in the current work.

In the future, we want to test the proposed model using different datasets and refine its hyperparameters via grid search. Additionally, accuracy can be improved by using optimization algorithms. Additionally, we intend to evaluate how well our models perform when used with AD, cognitively normal (NC), and mild cognitive impairment (MCI).

## Figures and Tables

**Table 1:** Representation of CDR value.

CDR	Risk Factor
No dementia	0
Very light dementia	0.5
Light dementia	1
Moderate dementia	2

**Table 2:** System components and its specification.

System Components	Description
Operating system	Windows 10 64 bit
CPU	Intel(R) Core(TM) i5-8500K CPU @ 3.70 GHz
RAM	16 GB
Programing language	Python 3.7.11
Tensorflow	Tensorflow version 2.6.0
IDE	jupyter

**Table 3:** Comparison of the classification of AD using different models.

Methods	Accuracy	Sensitivity	Specificity	AUC
SVM	0.93	0.93	0.94	0.94
KNN	0.91	0.87	0.88	0.91
Logistic Regression	0.87	0.86	0.87	0.87
Naive Bayes	0.78	0.73	0.83	0.78
XGBClassifier	0.77	0.74	0.78	0.77
Decision Tree	0.65	0.65	0.66	0.65
Random Forest	0.72	0.71	0.74	0.74
ANN [44]	0.89	-	-	-
Proposed model	0.96	0.94	0.96	0.97

**Table 4:** Comparison of AD classification with different ensemble classification methods.

Algorithm	Accuracy (%)	Sensitivity (%)	Specificity (%)	AUC (%)
Boosting (AdaBoost + DT)	90.00	82.65	89.76	91.40
Stacking (DT + KNN + LR)	83.33	80.84	83.85	84.77
SVM + MLP + J48 [45]	91.54	91.45	91.60	92.11
SVM + DT	90.43	90.11	89.66	91.45
Proposed Method	96.43	94.64	96.81	97.26

## Data Availability

Not applicable.

## Abbreviations

The following abbreviations are used in this manuscript:

AD	Alzheimer’s Disease
MRI	Magnetic Resonance Imaging
CAD	Computer-Aided Diagnostics
CT	Computed Tomography
PET	Positron Emission Tomography
OASIS	Open Access Series of Imaging Studies
CDR	Clinical Dementia Rating
MMSE	Mini-mental State Examination
SVM	Support vector machine
SCF	Cerebrospinal Fluid
WM	White Matter
GM	Gray Matter
KNN	K nearest neighbor
DT	Decision Tree
RF	Random Forest
LR	Logistic Regression
AUC	Area under the curve
ROC	Receiver operating characteristics
MCI	Mild Cognitive Impairment
NC	Cognitively Normal
MP-RAGE	Magnetization Prepared Rapid Gradient-Echo
